# Machine Learning Model for Predicting Suicide Risks Among Patients With Posttraumatic Stress Disorder Who Received Opioids

**DOI:** 10.1155/da/5305506

**Published:** 2026-07-30

**Authors:** Shu Huang, Amie J. Goodin, Jill A. Star, Serena Jingchuan Guo, Jiang Bian, Jie Xu, Tianze Jiao

**Affiliations:** ^1^ Department of Pharmaceutical Outcomes and Policy, College of Pharmacy, University of Florida, Gainesville 32611, Florida, USA, ufl.edu; ^2^ Center for Drug Evaluation and Safety (CoDES), College of Pharmacy, University of Florida, Gainesville 32611, Florida, USA, ufl.edu; ^3^ Department of Psychiatry, College of Medicine, University of Florida, Gainesville 32611, Florida, USA, ufl.edu; ^4^ Department of Biostatistics and Health Data Science, School of Medicine, Indiana University, Indianapolis 46202, Indiana, USA, iu.edu; ^5^ Regenstrief Institute, School of Medicine, Indiana University, Indianapolis 46202, Indiana, USA, iu.edu; ^6^ Department of Health Outcomes and Biomedical Informatics, College of Medicine, University of Florida, Gainesville 32611, Florida, USA, ufl.edu

**Keywords:** machine learning, opioids, posttraumatic stress disorder, suicide prediction

## Abstract

**Background:**

The cooccurrence of posttraumatic stress disorder (PTSD) and opioid use heightens suicide risk. We aimed to develop and validate a machine learning‐based suicide prediction model (SPM) to identify PTSD patients prescribed opioids who are at risk of suicide within 6‐month prediction intervals.

**Methods:**

Using 2016–21 OneFlorida+ data, we compared the predictive performance of multiple models, including least absolute shrinkage and selection operator (LASSO) regression, gradient boosting machines (GBMs), random forest (RF), and deep neural networks (DNNs). The best‐performing SPM was selected to predict 6‐month suicide risks among adult PTSD patients who received opioids. The index date was the first day when both an opioid prescription and a PTSD diagnosis occurred within 180 days. We divided the 2016–18 cohort into training and internal validation datasets (2:1 ratio), applying machine learning models to the training dataset to predict suicide‐related outcomes. We evaluated model prediction performance using various metrics on internal (2016–18) and external (2019–21) validation cohorts.

**Results:**

Among 5578 patients (age = 38.1 ± 11.1, female = 83.2%) in the 2016–18 cohort, 709 (12.7%) patients had at least one suicide‐related outcome during follow‐up. The final RF model selected 271/299 covariates, with C‐statistics, accuracy, sensitivity, specificity, and precision being 83.6% (81.7%−85.9%), 86.2% (85.2%−87.1%), 65.0% (59.8%−70.1%), 87.6% (86.7%−88.5%), and 26.6% (24.6%−28.8%), respectively. We classified intervals into 10 suicide‐risk subgroups based on the predicted probabilities, with 79.2% of suicide intervals captured in the top 3 decile subgroups. We observed similar findings in the 2019–21 cohort (*n* = 4849), with C‐statistics, accuracy, sensitivity, specificity, and precision being 83.6% (82.2%−84.9%), 87.0% (86.5%−87.6%), 65.3% (61.7%−68.7%), 88.2% (87.6%−88.7%), and 22.3% (21.1%−23.5%), respectively.

**Conclusions:**

Our SPM performed well in internal and external validations. It may serve as a feasible tool to identify patients at risk of suicide, helping prioritize preventive interventions, and enabling providers to allocate time and resources more efficiently to patients who may benefit from closer follow‐up.

## 1. Introduction

Suicide is a leading cause of death in the United States (US) and around the world [1, 2]. In 2023, suicide claimed over 49,300 lives in the US [2, 3]. Recognizing the multifaceted nature of suicide behavior (SB), including fatal and nonfatal self‐harm actions, is crucial for implementing effective interventions [4, 5]. Generally, SB includes suicide ideation (SI), suicide attempts (SA), and suicide death (SD) [6, 7]. SI/SA are behavioral outcomes that can repeatedly occur, increasing the risk of ultimate SD. The consequences of SB extend beyond physical harm, affecting psychological and social well‐being while placing substantial burdens on healthcare, social, and economic systems [2, 8].

It is estimated that 3.5%−4.7% of individuals experience posttraumatic stress disorder (PTSD) annually in the US [9, 10]. The lifetime prevalence of PTSD is estimated to be 6.8% [11]. However, this rate is higher among those with chronic pain (pooled mean prevalence ~10%), varying by the type of pain and severity [12, 13]. Veterans with pain are particularly at risk, with PTSD rates reaching as high as 50.1% [12].

The interplaying relationship between pain management, opioid use disorders (OUD), PTSD, and suicide is complex [14, 15]. PTSD patients with pain conditions face an increased risk of suicide, likely stemming from psychological burdens of PTSD and opioid misuse due to challenges in pain management [1, 16–19]. Evidence shows that PTSD patients have higher rates of opioid use, both of which have synergistic effects on worsening symptoms and expediting to negative outcomes, including OUD and suicide [9, 18, 20, 21]. In addition, OUD, PTSD, and suicide share common risk factors, such as pain and depression, with OUD being a major contributor to opioid overdose and SD [18, 22, 23].

Suicide prediction models (SPMs) could identify at‐risk individuals and prevent suicide‐related outcomes for PTSD patients [24, 25]. However, current SPMs mainly focus on the general population [26, 27], with low precision due to the rarity of events [28, 29], restricting clinical usefulness given the substantial frequency of false positives. One study developed an SPM with high precision (>80%) for PTSD patients with bipolar disorder using electronic health records (EHR) [30]. However, this study did not consider medication effects by class, excluded several key comorbidities, and did not conduct external validations, limiting the generalizability and robustness of findings. Additionally, the study identified suicide‐related outcomes from all settings, which may overfit models based on historical events instead of identifying incident events.

SPMs use traditional statistical models requiring specific structural assumptions (e.g., linearity), which might not hold due to the complex psychological and etiological framework of the SB and real‐world practice. Indeed, SB is a complex behavior, resulting from dynamic, nonlinear interactions among multiple psychological, clinical, social, and biological factors; relying on linear assumptions may fail to capture its risk patterns [4, 31]. In contrast, machine learning algorithms could handle nonlinear and nonmonotone associations, improving the accuracy of predictive models [32–34]. We hypothesize that machine learning‐based SPMs would outperform traditional statistical methods in predicting short‐term suicide risk and would more efficiently distinguish patients at elevated risk from others. The objective of our study is to develop and validate a machine learning‐based SPM to predict suicide‐related outcomes among PTSD patients who received opioids using large real‐world data.

## 2. Methods

This prognostic study followed the Transparent Reporting of a Multivariable Prediction Model for Individual Prognosis or Diagnosis reporting guidelines [29]. This study was exempted by the University of Florida Institutional Review Board (IRB202101897).

### 2.1. Data Sources

We used datasets from OneFlorida+ (OneFL) data trust, including 2016–21 Florida Medicaid claims data linked with OneFL EHR. OneFL is a centralized clinical research network covering ~75% of Floridians across 67 Florida counties [35] and the southeastern US [35]. About 3.6 million people were enrolled in Florida Medicaid in 2024, accounting for ~15% of the state population [36]. The linked data provides a comprehensive overview of patients’ medical and prescription histories.

### 2.2. Study Design and Cohort

This is a retrospective cohort study. The index date was defined as the first day when both an opioid prescription and a PTSD diagnosis occurred within 180 days. We applied the 180‐day gap, considering the delayed diagnosis of PTSD, given that opioid prescription may induce a PTSD flare [37] or increased hesitation to pursue healthcare based on the pattern in our data [38]. We focused on opioids for pain management: excluding intravenous and injectable opioids administered in inpatient (IP) settings [39], cough/cold medications containing opioids, and buprenorphine formulations indicated for OUD [40].

We created two cohorts for model development and external validation using data from 2016 to 18 and 2019–21, respectively. We excluded patients who (1) didn’t have the linked data, (2) never received opioid prescriptions or been diagnosed with PTSD, (3) were aged <18 at the index date, (4) had malignant cancer diagnoses or were in hospice, (5) didn’t have continuous Medicaid enrollment for ≥6 months after the index date, and (6) had an index date after July 1^st^, 2018 and July 1^st^, 2021 respectively (insufficient follow up) in each cohort, respectively.

**Figure 1:**
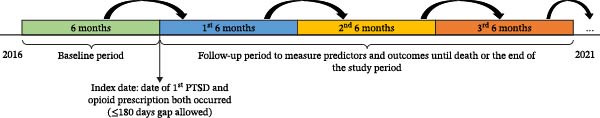
Suicide prediction modeling study design schematic diagram. PTSD, posttraumatic stress disorder.

We used a 6‐month baseline period to measure patients’ demographics and medical history before the index date (Figure 1). We divided the follow‐up period into several 6‐month intervals to measure predictors and subsequent outcomes. Predictors measured in the preceding interval were used to predict the outcomes in the subsequent interval. The 6‐month interval allows sufficient time to capture outcomes with a strong temporal connection to predictors, especially for SI/SA, behavioral outcomes that can repeatedly occur [41]. Patients remained in the cohort and were censored when having suicide‐related outcomes, died (other than SD), discontinued enrollment from Medicaid, or had no EHR encounters for >6 months or at the end of the study.

### 2.3. Measurement of Outcome

The primary outcome was a composite suicide‐related outcome: SI, SA, or SD determined by International Classification of Diseases Clinical Modification (ICD‐10‐CM) codes in IP or emergency department (ED) visits (eTable 1) [42]. Recent studies show that these ICD‐10‐CM diagnosis codes have high precision (>83%) in different populations [43–45]. Suicide outcomes that occurred in IP/ED could better capture incident cases of patients at acute risk who need timely intervention [46–48]. This composite definition was chosen to capture clinically significant SB that prompts acute care utilization and reflects elevated short‐term risk.

### 2.4. SPM Predictors

Based on the literature review and clinical experience, we identified 299 predictors (eTable 2), including patient‐level sociodemographic factors (e.g., age, gender, race, ethnicity, and rurality of residence), provider‐level characteristics (e.g., specialty), regional‐level characteristics (e.g., area deprivation index), clinical factors (e.g., diagnoses, procedures), and prescriptions during each of the 6‐month intervals [27, 49]. Publicly available sources (e.g., area health resource files) were used and linked with patients’ zip codes to generate regional‐level characteristics.

We applied a mixed approach to address data missingness. For incurable/chronic diseases (e.g., family history of mental and behavioral disorders, epilepsy, and bipolar disorder), once the patient was diagnosed, we carried the last observation forward throughout intervals. For curable/acute conditions (e.g., dizziness and fatigue), we changed the missing to zero, as these conditions may have been treated/resolved in the current interval (eTable 3). For opioid dose measurements and regional variables (e.g., average opioid daily dose in Morphine Milligram Equivalent [MME], regional homicide rate), we replaced missing values with the mean in the existing 6‐month interval to preserve the overall data distribution. We additionally excluded predictors with >50% missingness.

We explicitly accounted for the temporal variation of suicide‐related behaviors and clinical characteristics. Suicide history and related clinical events were measured in a time‐varying manner across predefined 6‐month intervals rather than as a static time‐independent variable. This design allows more recent events to be distinguished from distant ones, aligning with clinical reasoning that recent SI/SA carries greater prognostic significance than the remote history.

### 2.5. Statistical Analysis

Our machine learning analysis comprised two steps: (1) developing an SPM model and creating suicide risk prediction scores and (2) stratifying intervals into subgroups with similar suicide risks.

We randomly divided the 2016–18 cohort into training and testing datasets with a 2:1 ratio, treating the testing data as internal validation. We then externally validated the selected SPM using the 2019–21 cohort. We treated the repeated measurements in different time intervals as independent, assuming machine learning methods were more flexible with highly correlated observations. We used Python 3.8 to compare four SPM models, including least absolute shrinkage and selection operator (LASSO) regression, gradient boosting machine (GBM), random forest (RF), and deep neural networks (DNN). These models were intentionally selected to represent complementary methodological paradigms with varied assumptions, levels of complexity, and strengths, allowing for a principled comparison rather than arbitrarily selecting one. LASSO regression was included as a regularized linear model that performs embedded feature selection and offers high interpretability, serving as a strong and commonly used foundational predictive model in clinical research. GBM and RF were selected as ensemble tree‐based methods capable of capturing complex nonlinear relationships and interactions among predictors, which are expected in SPM studies. RF provides robustness and stability, whereas GBM allows for more flexible, sequential error correction. Finally, DNN was included to assess whether highly expressive models can leverage potential higher‐order patterns in the data beyond those captured by traditional and ensemble methods. Together, this set of models spans a continuum from interpretable linear approaches to highly flexible nonlinear methods, enabling us to evaluate the trade‐off between interpretability and predictive performance with different assumptions in a clinically relevant context.

According to a recent systematic review, RF and support vector machine (SVM) were the most used machine learning SPMs in psychiatric populations [50]. In our study, RF was selected specifically due to its suitability for the characteristics of our data (high‐dimensional factors highly correlated with complex nonlinear relationships) and research goals. In addition, RF is relatively robust to overfitting, requires limited feature preprocessing, and provides measures of feature importance, which support the interpretability objectives of this study. While the performance of SVM is highly sensitive to kernel choice and hyperparameter tuning and could not provide interpretable feature importance measures by design. Given our emphasis on model stability and interpretability alongside predictive performance, RF was therefore deemed more appropriate for the present analysis.

To address class imbalance without artificially altering the outcome distribution, we considered and applied several model engineering strategies. First, we preserved the original event rate in all analyses and did not generate synthetic samples or rebalance the dataset to a 50/50 distribution. This decision was made to maintain clinical realism and avoid inflating predictive performance. Instead, we adopted metric‐driven and validation‐based approaches that are robust to class imbalance. Using the training dataset, we performed fivefold cross‐validation and tested different hyperparameters to optimize the C‐statistics of each model. Using the testing dataset, we evaluated the model performance via several metrics (e.g., sensitivity, specificity, and F‐1 score), and the model with the best prediction performance was selected as the final model (eTable 4). We also reported the top 25 predictors, the receiver operating characteristic (ROC) curve, and the precision–recall curve [51]. We applied decile thresholds for the predicted risk of suicide‐related outcomes derived from the training dataset to facilitate comparison of risk profiles in the testing dataset. To test the external validity, we applied the same model to the 2019–21 cohort and evaluated its prediction performance and risk stratification using the same metrics mentioned above.

We conducted several sensitivity analyses. First, we varied the length of the outcome measurement from 6 to 3 months to examine whether shorter intervals could capture more immediate changes in suicide‐related outcomes. Second, we additionally captured suicide‐related outcomes that occurred during outpatient visits to examine the reliability of the outcome. Third, previous studies showed racial disparities in the treatment effects of opioid use [52], which may impact the performance of our model. Thus, we evaluated the race‐stratified false‐negative and false‐positive rates and additionally reported ROC curves, precision‐recall curves, and calibration plots by race. Fourth, we assessed the clinical utility of the selected model through decision curve analysis.

## 3. Results

**Figure 2:**
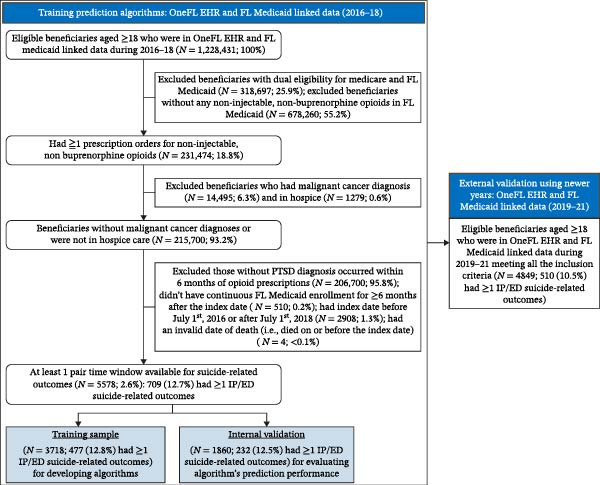
Flow chart of the study cohorts. ED, emergency department; EHR, electronic health record; FL, florida; IP, inpatient.

Among 5578 patients in the 2016–18 cohort (mean age: 38.1 ± 11.1, female: 83.2%, and white patients: 69.1%), 709 (12.7%) patients experienced ≥1 outcome during follow‐up. Patients in training (*n* = 3718) and testing (*n* = 1860) datasets of the 2016–18 cohort had similar baseline characteristics and outcome distributions (Table 1 and Figure 2). Compared with the overall 2016–18 cohort, the 2019–21 cohort (*n* = 4849) was slightly older (38.9 ± 11.3 vs. 38.1 ± 11.1); less likely to be White patients (68.8% vs. 69.1%), reside in the metropolitan area (90.7% vs. 91.4%), have treated PTSD (33.6% vs. 34.2%), or receive antidepressants (60.6% vs. 61.7%); sicker (Elixhauser Comorbidity Index [ECI]: 2.9 vs. 2.7), have obesity (37.2% vs. 28.5%), anxiety (64.7% vs. 60.9%), abdominal pain (48.0% vs. 46.4%), neck pain (24.3% vs. 21.9%), or osteoarthrosis (34.6% vs. 30.0%). The suicide‐related outcome rate was lower in the 2019–21 (10.5%) than in the 2016–18 cohort (12.7%).

**Table 1:** Selected baseline characteristics of OneFL EHR and Florida Medicaid linked data (patient level).

Baseline characteristics	OneFL 2016–18 EHR and Florida Medicaid linked data			OneFL 2019–21 EHR and Florida Medicaid linked data
	Training (*n* = 3718)	Testing (*n* = 1860)	Overall (*n* = 5578)	Validation (*n* = 4849)
Age at index, mean (SD)	38.1 (11.1)	38.1 (11.0)	38.1 (11.1)	38.9 (11.3)
Any IP/ED suicide‐related outcomes in follow‐up	477 (12.8)	232 (12.5)	709 (12.7)	510 (10.5)
Any IP/ED/OP suicide‐related outcomes in follow‐up	517 (13.9)	254 (13.7)	771 (13.8)	551 (11.4)
Gender				
Female	3124 (84.0)	1518 (81.6)	4642 (83.2)	4070 (83.9)
Male	594 (16.0)	342 (18.4)	936 (16.8)	779 (16.1)
Race				
White	2587 (69.6)	1267 (68.1)	3854 (69.1)	3338 (68.8)
Black	830 (22.3)	426 (22.9)	1256 (22.5)	1133 (23.4)
Other	301 (8.1)	167 (9.0)	468 (8.4)	378 (7.8)
Ethnicity				
Hispanic	614 (16.5)	300 (16.1)	914 (16.4)	771 (15.9)
Nonhispanic	3014 (81.1)	1511 (81.2)	4525 (81.1)	3944 (81.3)
Unknown/missing	90 (2.4)	49 (2.6)	139 (2.5)	134 (2.8)
Health service use				
≥1 IP visit	1586 (42.7)	796 (42.8)	2382 (42.7)	2157 (44.5)
≥1 ED visit	3004 (80.8)	1533 (82.4)	4537 (81.3)	3815 (78.7)
≥1 OP visit	3654 (98.3)	1831 (98.4)	5485 (98.3)	4729 (97.5)
Metropolitan area	3402 (91.5)	1694 (91.1)	5096 (91.4)	4398 (90.7)
Medication use				
Any antidepressants use	2302 (61.9)	1141 (61.3)	3443 (61.7)	2937 (60.6)
Any buprenorphine use	54 (1.5)	20 (1.1)	74 (1.3)	100 (2.1)
Any benzodiazepine use	1557 (41.9)	800 (43.0)	2357 (42.3)	1645 (33.9)
Any gabapentinoids use	1064 (28.6)	509 (27.4)	1573 (28.2)	1544 (31.8)
Any muscle relaxants use	1357 (36.5)	645 (34.7)	2002 (35.9)	1905 (39.3)
Any naloxone use	53 (1.4)	19 (1.0)	72 (1.3)	236 (4.9)
Any naltrexone use	59 (1.6)	36 (1.9)	95 (1.7)	36 (0.7)
Any non‐IV opioid use	3718 (100.0)	1860 (100.0)	5578 (100.0)	4849 (100.0)
Any antitussive use	22 (0.6)	10 (0.5)	32 (0.6)	4 (0.1)
Any anti‐PTSD medication use	1195 (32.1)	583 (31.3)	1778 (31.9)	1439 (29.7)
Number of medications used, mean (SD)	14.3 (15.4)	13.5 (13.8)	14.0 (14.9)	15.3 (16.8)
Polypharmacy (≥3 medications)	3342 (89.9)	1660 (89.2)	5002 (89.7)	4377 (90.3)
Daily opioid MME, mean (SD)	39.8 (54.7)	37.2 (34.9)	38.9 (49.0)	36.5 (42.3)
Number of opioid prescribers, mean (SD)	3.6 (2.8)	3.5 (2.6)	3.5 (2.7)	3.3 (2.5)
Main opioid prescriber gender				
Male	2216 (59.6)	1116 (60.0)	3332 (59.7)	2783 (57.4)
Female	1137 (30.6)	568 (30.5)	1705 (30.6)	1570 (32.4)
Missing/unknown	365 (9.8)	176 (9.5)	541 (9.7)	496 (10.2)
Main opioid prescriber specialty				
Primary care	1127 (30.3)	568 (30.5)	1695 (30.4)	1531 (31.6)
ED	1004 (27.0)	503 (27.0)	1507 (27.0)	827 (17.1)
Pain/physical/occupational therapy/Chiropractic	296 (8.0)	121 (6.5)	417 (7.5)	451 (9.3)
Surgery	250 (6.7)	131 (7.0)	381 (6.8)	442 (9.1)
Other/unknown	686 (18.5)	370 (19.9)	1056 (18.9)	1136 (23.4)
Elixhauser comorbidities^a^				
Elixhauser comorbidity index, mean (SD)	3.6 (2.7)	3.6 (2.7)	3.6 (2.7)	4.0 (2.9)
Congestive heart failure	124 (3.3)	78 (4.2)	202 (3.6)	247 (5.1)
Valvular disease	186 (5.0)	102 (5.5)	288 (5.2)	239 (4.9)
Pulmonary circulation disease	57 (1.5)	34 (1.8)	91 (1.6)	84 (1.7)
Peripheral vascular disease	134 (3.6)	79 (4.2)	213 (3.8)	238 (4.9)
Paralysis	72 (1.9)	42 (2.3)	114 (2.0)	152 (3.1)
Neurological disorders	717 (19.3)	356 (19.1)	1073 (19.2)	1002 (20.7)
Chronic pulmonary disease	1147 (30.8)	557 (29.9)	1704 (30.5)	1559 (32.2)
Diabetes without chronic complications	542 (14.6)	263 (14.1)	805 (14.4)	698 (14.4)
Diabetes with chronic complications	353 (9.5)	165 (8.9)	518 (9.3)	548 (11.3)
Hypothyroidism	318 (8.6)	176 (9.5)	494 (8.9)	463 (9.5)
Renal failure	99 (2.7)	63 (3.4)	162 (2.9)	180 (3.7)
Liver diseases	322 (8.7)	158 (8.5)	480 (8.6)	493 (10.2)
Peptic ulcer disease	69 (1.9)	41 (2.2)	110 (2.0)	126 (2.6)
Acquired immune deficiency syndrome	85 (2.3)	54 (2.9)	139 (2.5)	88 (1.8)
Rheumatoid arthritis	250 (6.7)	130 (7.0)	380 (6.8)	365 (7.5)
Coagulopathy	116 (3.1)	57 (3.1)	173 (3.1)	181 (3.7)
Obesity	1087 (29.2)	505 (27.2)	1592 (28.5)	1802 (37.2)
Weight loss	115 (3.1)	75 (4.0)	190 (3.4)	204 (4.2)
Fluid and electrolyte disorders	586 (15.8)	304 (16.3)	890 (16.0)	935 (19.3)
Chronic blood loss anemia	172 (4.6)	87 (4.7)	259 (4.6)	359 (7.4)
Deficiency Anemias	654 (17.6)	302 (16.2)	956 (17.1)	1015 (20.9)
Alcohol abuse	340 (9.1)	207 (11.1)	547 (9.8)	434 (9.0)
Drug abuse	907 (24.4)	445 (23.9)	1352 (24.2)	1188 (24.5)
Psychoses	1619 (43.5)	749 (40.3)	2368 (42.5)	1934 (39.9)
Depression	2011 (54.1)	1029 (55.3)	3040 (54.5)	2663 (54.9)
Hypertension	1433 (38.5)	723 (38.9)	2156 (38.7)	2020 (41.7)
Mental conditions				
PTSD	3718 (100.0)	1860 (100.0)	5578 (100.0)	4849 (100.0)
Treated PTSD	1282 (34.5)	624 (33.5)	1906 (34.2)	1628 (33.6)
Anxiety disorder	2279 (61.3)	1119 (60.2)	3398 (60.9)	3136 (64.7)
Mood Disorders	2613 (70.3)	1300 (69.9)	3913 (70.2)	3379 (69.7)
Personality disorders	267 (7.2)	128 (6.9)	395 (7.1)	283 (5.8)
Cognitive confusion	322 (8.7)	172 (9.2)	494 (8.9)	439 (9.1)
Delusional disorders	73 (2.0)	30 (1.6)	103 (1.8)	109 (2.2)
Bipolar disorder	1291 (34.7)	601 (32.3)	1892 (33.9)	1538 (31.7)
Symptoms and signs involving emotional state	478 (12.9)	240 (12.9)	718 (12.9)	523 (10.8)
Schizophrenic disorders	308 (8.3)	158 (8.5)	466 (8.4)	328 (6.8)
Nonorganic psychosis	310 (8.3)	162 (8.7)	472 (8.5)	333 (6.9)
Pain conditions				
Abdominal pain	1750 (47.1)	839 (45.1)	2589 (46.4)	2329 (48.0)
Back pain	1671 (44.9)	827 (44.5)	2498 (44.8)	2158 (44.5)
Chest pain	1055 (28.4)	535 (28.8)	1590 (28.5)	1384 (28.5)
Headache/migraine	1096 (29.5)	531 (28.5)	1627 (29.2)	1378 (28.4)
Neck pain	813 (21.9)	410 (22.0)	1223 (21.9)	1178 (24.3)
Menstrual/genital reproductive pain (females)	679 (18.3)	316 (17.0)	995 (17.8)	877 (18.1)
Kidney stones/gall stones	265 (7.1)	118 (6.3)	383 (6.9)	392 (8.1)
Fibromyalgia	465 (12.5)	221 (11.9)	686 (12.3)	606 (12.5)
Osteoarthrosis	1102 (29.6)	570 (30.6)	1672 (30.0)	1680 (34.6)
Other pain conditions	1317 (35.4)	688 (37.0)	2005 (35.9)	1859 (38.3)
Other comorbidities				
Suicide history (any clinical setting)^c^	469 (12.6)	239 (12.8)	708 (12.7)	483 (10.0)
Personal history of self‐harm^c^	63 (1.7)	39 (2.1)	102 (1.8)	159 (3.3)
Opioid‐related overdose	35 (0.9)	20 (1.1)	55 (1.0)	38 (0.8)
Opioid‐related adverse events (selected)^b^	1203 (32.4)	573 (30.8)	1776 (31.8)	1677 (34.6)
Drug overdose	293 (7.9)	146 (7.8)	439 (7.9)	354 (7.3)
Substance use disorder	2020 (54.3)	1012 (54.4)	3032 (54.4)	2663 (54.9)
Nonopioid substance use disorders	774 (20.8)	384 (20.6)	1158 (20.8)	926 (19.1)
Mental or behavioral disorders due to psychoactive substance use	1991 (53.6)	994 (53.4)	2985 (53.5)	2637 (54.4)
Alcohol use disorder	325 (8.7)	205 (11.0)	530 (9.5)	436 (9.0)
Cannabis use problems	456 (12.3)	230 (12.4)	686 (12.3)	674 (13.9)
Dizziness	384 (10.3)	186 (10.0)	570 (10.2)	474 (9.8)
Epilepsy	319 (8.6)	156 (8.4)	475 (8.5)	416 (8.6)
Musculoskeletal disorders	2515 (67.6)	1265 (68.0)	3780 (67.8)	3310 (68.3)
Respiratory diseases	1857 (49.9)	915 (49.2)	2772 (49.7)	2437 (50.3)
Ischemic heart disease	292 (7.9)	145 (7.8)	437 (7.8)	455 (9.4)
Stroke	58 (1.6)	36 (1.9)	94 (1.7)	126 (2.6)
Traumatic brain injury	88 (2.4)	50 (2.7)	138 (2.5)	126 (2.6)
Counseling	1440 (38.7)	722 (38.8)	2162 (38.8)	1909 (39.4)
Lost‐time injuries or major surgeries	438 (11.8)	236 (12.7)	674 (12.1)	838 (17.3)
Urine drug test	1269 (34.1)	632 (34.0)	1901 (34.1)	1995 (41.1)

*Note:* Data are *n* (%) or mean (SD).

Abbreviations: ED, emergency department; EHR, electronic health records; IP, inpatient; OP, outpatient; PTSD, posttraumatic stress disorder; SD, standard deviation.

^a^We excluded metastatic cancers, solid tumors with or without metastasis, and diagnoses specified individually from our Elixhauser Comorbidity Index score calculation because we only included noncancer patients.

^b^Opioid‐related adverse events (selected) include opioid‐induced sleep disorders, mental disorders, and respiratory issues. Opioid overdose includes nonfatal poisoning by heroin, methadone, opiates, and related narcotics. Nonopioid substance use disorders include nonfatal poisoning and disorders caused by substances other than opioids (e.g., alcohol, cannabis, etc.).

^c^Suicide history (any clinical setting) included suicide attempts, suicide ideation, and suicide death (eTable 1). Personal history of self‐harm was defined by the ICD‐10‐CM code Z91.5.

There were 10,918, 5356, and 15,039 episodes of 6‐month intervals in the training, testing, and external validation datasets, respectively. On average, each patient contributed to the prediction three times (eTable 5). The suicide‐related outcome rate was the highest in the first 6‐month interval (ranged 6.2%−8.3%). Since most patients didn’t have visits by the end of the study, loss of follow‐up led to a decline in the outcome rate. Specifically, patients may discontinue engagement in the OneFL system due to relocation to other states or discontinue their health insurance.

**Figure 3:**
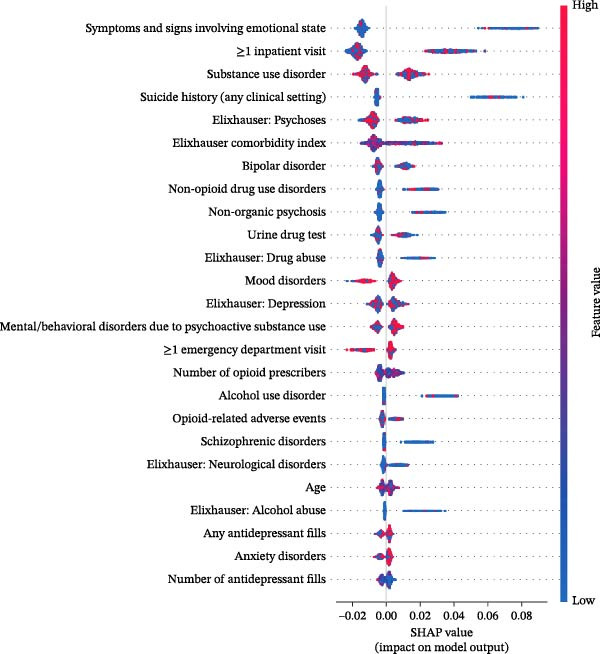
SHAP plot of the top 25 important predictors for suicide‐related outcomes in 2016–18 OneFL EHR and Florida Medicaid linked data selected by random forest.

After comparing four models, RF was selected because it was efficient and had better prediction performances: the highest F‐1 score, weighted accuracy, and the second highest C‐statistic (eTable 6). However, all models exhibited low precision and overfitting, likely due to the low event rate. RF performed well in internal and external validation datasets (C‐statistics: 2016–18: 83.6% [95%CI: 81.7%−85.9%] vs. 2019–21: 83.6% [82.2%−84.9%]) (eFigure 1). The final RF model included 271 out of 299 predictors, and the top five included symptoms and signs involving emotional state, ≥1 IP visit, substance use disorder (SUD), suicide history, and psychoses (Figure 3). The specificity, sensitivity, precision, accuracy, weighted accuracy, and F‐1 score were 87.6% (86.7%−88.5%), 65.0% (59.8%−70.1%), 26.6% (24.6%−28.8%), 86.2% (85.2%−87.1%), 81.8% (80.9%−82.7%), and 0.38; and 88.2% (87.6%−88.7%), 65.3% (61.7%−68.7%), 22.3% (21.1%−23.5%), 87.0% (86.5%−87.6%), 83.5% (82.9%−84.0%), and 0.33 in the 2016–18 and 2019–21 cohort, respectively.

**Figure 4:**
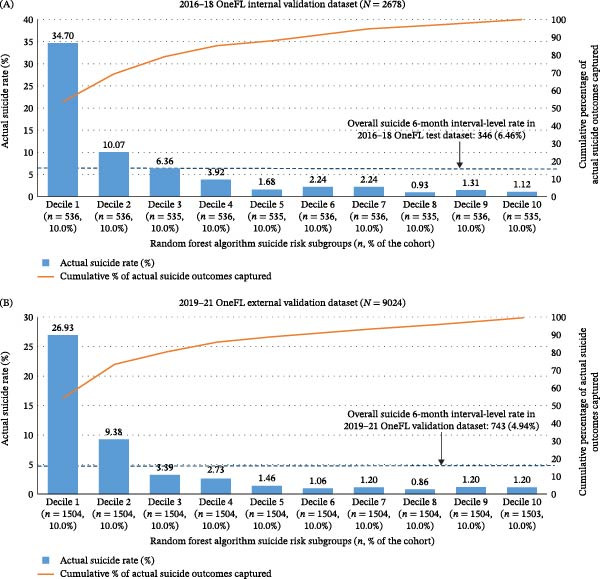
The 6‐month suicide‐related outcomes identified by risk subgroups in the OneFL internal and external validation datasets using random forest. (A) 2016–18 OneFL internal validation dataset (*N* = 2678), (B) 2019–21 OneFL external validation dataset (*N* = 9024). Based on the 6‐month interval’s predicted probability of suicide‐related outcomes from inpatient or emergency department settings, we classified intervals in the two validation datasets into risk subgroups using the decile thresholds of the risk scores derived from the 2016–18 OneFL algorithm development (training) dataset. The thresholds of the risk scores derived from the 2016–18 OneFL training dataset to identify suicide risk subgroups are: decile 1 (risk score ≥ 58.3); decile 2 (41.8 ≤ risk score < 58.3); decile 3 (33.9 ≤ risk score < 41.8); decile 4 (28.4≤ risk score < 33.9); decile 5 (24.4 ≤ risk score < 28.4); decile 6 (21.1 ≤ risk score < 24.4); decile 7 (18.5 ≤ risk score < 21.1); decile 8 (15.9 ≤ risk score < 18.5); decile 9 (12.6 ≤ risk score < 15.9); and decile 10 (risk score < 12.6).

We classified the cohort into 10 suicide‐risk subgroups based on the predicted probabilities of suicide‐related outcomes (Figure 4). Among 5356 intervals in the 2016–18 testing dataset, 346 (6.5%) had outcomes. Notably, ~80% (*n* = 274) of these intervals were captured in the top three deciles of risk subgroups with outcome rates ranging 6.4%−34.7%. The highest‐risk subgroup (Decile 1: *n* = 536) had a precision of 34.7%. The overall suicide‐related outcomes rate was lower in the 2019–21 cohort (4.9% vs. 6.5%), with 597 (80.4%) intervals correctly captured in the top three deciles of risk subgroups with event rates ranging 3.4%−26.9%. The highest‐risk subgroup (Decile 1: *n* = 1504) had a precision of 26.9%. The calibration plot indicates that our RF model tends to overestimate suicide risks (eFigure 2).

The sensitivity analyses demonstrated the robustness of our findings. When switching to 3‐month follow‐up intervals, the C‐statistic was improved from 83.6% to 85.4% and 85.1% in 2016–18 and 2019–21 cohorts, respectively. For the 2016–18 testing dataset, the specificity, sensitivity, precision, accuracy, and F‐1 score were similar as the 6‐month model: 87.7% (87.1%−88.3%), 68.9% (64.2%−73.2%), 17.8% (16.7%−19.0%), 87.0% (86.4%−87.6%), and 0.28, respectively. Second, after including suicide‐related outcomes from outpatient visits, the results were comparable to the primary analysis. The C‐statistic slightly decreased (2016–18: 82.8% [80.9%−85.0%] and 2019–21: 82.6% [81.2%−84.1%]). For the 2016–18 testing dataset, the specificity, sensitivity, precision, accuracy, and F‐1 score were 87.6% (86.7%−88.5%), 64.3% (59.2%−69.2%), 27.8% (25.8%−30.0%), 86.0% (85.1%−86.9%), and 0.39, respectively. Thirdly, we evaluated multiple, potentially competing criteria rather than a single metric for the race fairness evaluation. False negative and false positive rates, C‐statistics, ROC curves, precision‐recall curves, and calibration plots between Black and White patients were comparable in both cohorts, suggesting a low risk of race discrimination in the RF model (eFigure 3). These findings indicate a balanced trade‐off between error rates and predictive accuracy across racial groups, in line with current fairness guidelines. Fourth, the decision curve analysis demonstrated that the RF model provides meaningful clinical utility across threshold probabilities ranging from ~1% to 60% (eFigure 4). Across this range, the model’s net benefit remains consistently positive and exceeds that of both treat‐all and treat‐none strategies. At lower thresholds (e.g., 1%–5%), which are particularly relevant in suicide prevention where missing a high‐risk individual carries substantial consequences, the model yields the greatest net benefit. This indicates that using the model to guide intervention decisions would identify more individuals at true risk of suicide while avoiding unnecessary interventions compared with a strategy of intervening on all individuals. Overall, it suggests that implementing the RF model to guide suicide prevention efforts could improve clinical decision‐making across a broad and clinically plausible range of risk thresholds, supporting its potential value for targeted intervention, enhanced close monitoring, and reallocation of limited health resources.

## 4. Discussion

We developed and validated an RF algorithm with good performance for predicting 6‐month risks of IP/ED suicide‐related outcomes in PTSD patients who received opioids. The RF model identified variables, including suicide history, SUD, comorbid depression, alcohol use disorder, and prior mental health symptoms, as significant predictors, reflecting both psychological and physiological contributions to SB. It illustrates that computational approaches can complement existing theory by uncovering latent risk factors and patterns, thereby enhancing early identification and informing targeted interventions.

Our study showed that an SPM based on a state’s real‐world data can effectively predict 6‐month suicide‐related outcomes. When validating the SPM using external data from more recent years with a population of different characteristics, the model demonstrated robust findings, supporting its generalizability across different timeframes and patient groups. Our RF algorithm represented an improvement by including a comprehensive list of predictors, enabling a more robust assessment of suicide risk than traditional models [30]. Specifically, our model incorporated detailed disease diagnoses, opioid use in daily MME, prescriber‐level characteristics, and regional‐level factors.

We applied multiple performance metrics when evaluating an SPM. For example, relying solely on C‐statistics can be misleading when the event rate is low. Although our model showed good discrimination (C‐statistics > 0.80), rare suicide‐related outcomes led to low precision that could increase false positives. Thus, we reported sensitivity, weighted accuracy, F‐1 score, and risk subgroup analysis to provide thorough evaluations. Given the severe consequences of suicide‐related outcomes, missing a high‐risk patient (false negative) is much more dangerous than incorrectly flagging a low‐risk patient (false positive). We calculated a weighted accuracy, assigning a penalty to false‐negative events five times greater than that to false‐positive events, as determined by clinical experts [53]. While our RF model showed acceptable sensitivity and a relatively low F‐1 score, it achieved high weighted accuracy, with ~80% of suicide events captured in the top three deciles of risk subgroups. Although our model exhibits signs of overfitting, in the context of highly imbalanced datasets and the catastrophic consequences of suicide‐related events, this trade‐off is acceptable from a benefit–harm perspective. The cost of missing a suicide‐related outcome is extremely high; it is crucial to prioritize sensitivity and reduce the risk of false negatives to identify as many true positives as possible in suicide screening. While high sensitivity can improve clinical detection, it may increase false positives; therefore, continuous close monitoring and recalibration can mitigate the risks of overfitting while preserving fairness and clinical utility over time.

Our analysis indicated that patients with mental disorders (e.g., symptoms involving emotional state and depression), IP visits, suicide history, and SUD were at elevated risks of suicide‐related outcomes in the following 6‐month interval. Patients suffering from more comorbidities (e.g., higher ECI) were more likely to experience suicide‐related outcomes [27, 30]. Although using the suicide history to predict future suicide‐related outcomes may seem self‐evident, individuals exhibiting these behaviors are often in an acute psychological state (e.g., stress disorder), making them vulnerable to taking immediate action. Furthermore, SB severity varies and can evolve rapidly. Capturing history may help enable timely and appropriate interventions to prevent tragic outcomes. Our model selected most predictors; however, it doesn’t imply that the unselected predictors (e.g., naltrexone use, buprenorphine use, and tinnitus) were not useful. The feature selection process aimed to identify a balanced set of predictors comprehensive enough to ensure accurate predictions while minimizing redundancy and to optimize model performance. When implementing our SPM for suicide screening, it’s crucial to include the top‐ranked risk factors, if not all, that are measurable in the data.

Among risk stratification subgroups, we observed low suicide‐related outcomes in Deciles 5–10 with <20 cases each (Figure 4). This limited sample size may have contributed to the reverse suicide‐related outcome rates in Deciles 5–6. When events are few, small data fluctuations can significantly impact model performance and outcome distribution, making it challenging to conclude risk stratification in these deciles.

When adjusting the outcome measurement interval to 3 months, the RF model demonstrated a slight improvement in C‐statistic, sensitivity, specificity, and accuracy. This was primarily due to the increased number of intervals, which provided more data points for model training and validation, potentially enhancing the ability to learn patterns associated with suicide‐related risks. However, since the rate of 3‐month suicide‐related outcomes decreased, precision and the F‐1 score were lower as these metrics are sensitive to outcome prevalence. This trade‐off highlights the balance between capturing more granular temporal relationships and maintaining predictive performance in suicide risk assessments.

We identified suicide‐related outcomes via IP/ED visits in the primary analysis as we would like to focus on severe events at acute risk. However, recognizing that outpatient management of PTSD would likely capture SI, although some might be historical, we expanded the scope to include outpatient suicide‐related outcomes in our sensitivity analyses. Despite this broader inclusion, the model performance remained robust, indicating that our approach could be effective in identifying both severe and moderate suicide risks from all clinical visits.

Our model advances current SPM strategies, allowing for more effective targeting of time‐sensitive interventions. The study showed that using different risk score thresholds (e.g., Decile 1) to identify high‐risk patients was accurate. Our risk classification method allows implementers to set the risk threshold for intervention, considering costs, intervention intensity, and available resources. Resource‐intensive interventions could be focused on individuals in the highest‐risk subgroup, while lower‐cost, less burdensome interventions could target those in moderate‐to‐high‐risk subgroups [54, 55]. Nonetheless, additional screening and assessment are needed to avoid unintended consequences resulting from false positives. In practice, the model is intended to support clinicians by helping prioritize close monitoring, targeted assessment, and preventive interventions, allowing providers to allocate limited time and resources more efficiently to patients who may benefit from closer monitoring. The probabilities or risk scores generated by the model should be carefully interpreted as relative risk indicators within a specific time horizon rather than as absolute predication or causal statements about future SB.

We acknowledge that alternative approaches, such as landmarking or discrete‐time survival analysis, may better account for within‐patient correlation and reduce potential pseudo‐replication. Compared with our temporal modeling framework (i.e., the 6‐month SPM, which leverages longitudinal data to update suicide risk dynamically), landmarking and discrete‐time survival analysis represent more statistically conservative strategies for handling repeated observations over time. Landmarking generates predictions from predefined time points and minimizes overlapping risk windows, while discrete‐time survival analysis estimates the probability of suicide within structured intervals and explicitly accounts for within‐person dependence and censoring. In contrast, temporal modeling frameworks often leverage fine‐grained time‐varying data and may capture complex trajectories of risk, but they can still be vulnerable to pseudo‐replication or overly optimistic performance if within‐person dependence is not properly handled. As a result, landmarking and discrete‐time survival models may yield lower discrimination but improved calibration, whereas temporal frameworks offer greater flexibility and potentially higher predictive performance at the cost of increased model complexity and risk of overfitting. Additionally, we believe that the temporal framework is better suited to address this clinically important question: whether patients are at elevated risk of suicide within the next 3 or 6 months at a given encounter, regardless of the timing and frequency of previous visits.

Our study has limitations. First, misclassification of SD may occur since OneFL is not fully linked to the death certificate. However, we measured a composite of SI/SA/SD using validated ICD‐10‐CM codes with high precision (>83%) [43–45]. Second, we did not extract predictors from unstructured data, including laboratory results and clinical notes [56]. These unstructured data often contain valuable clinical insights, including disease severity, physician assessments, and nuanced patient symptoms that structured data may not fully capture. However, much of this information was already inferred through the structured data. Incorporating unstructured data in future studies could further enhance the model’s performance. Integrating broader psychosocial and social determinant information into future models could also enhance predictive accuracy and promote a more holistic understanding of risk, consistent with human‐centered approaches to suicide prevention [31]. Future work should aim to validate the identified risk factors and their patterns and integrate additional psychosocial variables to refine both predictive accuracy and theoretical understanding of suicide risk in this vulnerable population. Third, we required patients to have ≥1 EHR encounter within 6‐month intervals, which implies that the model is capturing those with higher healthcare needs. Fourth, our prediction algorithm may not be generalizable to other populations or states as it is primarily based on a linked database of Floridians receiving Medicaid. Requiring continuous Medicaid enrollment may limit generalizability as individuals enrolled long‐term are likely to be in poorer health or have greater healthcare needs than those who are eligible for Medicaid but not continuously enrolled. Fifth, the precision was low due to the rarity of suicide‐related outcomes. Nonetheless, our risk‐stratified approach appeared to be effective in identifying the majority of the events (~80%), providing valuable insights for directing timely interventions. If applied to a more vulnerable patient population (e.g., veterans), the algorithm would potentially achieve better performance. Finally, key barriers need to be addressed before implementation, such as concerns about the usability and effectiveness of the algorithm due to data lags in EHR and claims data. However, our top 25 predictors were primarily derived from diagnoses, prescriptions, and encounter files, generally available for patients at regular visits. Beyond the top five predictors, the remaining exhibited relatively low and comparable importance scores, indicating the model’s performance wasn’t heavily reliant on any single covariate. This would enhance the model’s robustness and ensure its applicability across varying levels of data completeness. Additionally, the current algorithm includes race and ethnicity, requiring comprehensive bias evaluations to ensure algorithm fairness in target interventions and provide health services equitably.

## 5. Conclusions

This study developed and validated an RF algorithm to predict 6‐month suicide risks among PTSD patients prescribed opioids. This model has the potential for clinical application in identifying high‐risk patients to facilitate timely interventions. The model is designed to serve as a clinical decision‐support tool that identifies individuals at elevated short‐term (3‐ or 6‐month) risk, thereby raising a “red flag” to prompt closer clinical attention rather than replacing clinical judgment. Further external validation and thorough bias assessment are necessary before clinical implementation to ensure reliability and fairness.

## Author Contributions

The study design and analyses of the manuscript, as well as the decision to submit the manuscript for publication, were entirely completed by all authors.

## Funding

No funding was received for this research.

## Disclosure

All authors have reviewed and approved the manuscript.

## Conflicts of Interest

Shu Huang has received funding from Merck, Sharp, and Dohme, unrelated to this work. The remaining authors declare no conflicts of interest.

## Supporting Information

Additional supporting information can be found online in the Supporting Information section.

## Supporting information


**Supporting Information** The supplementary tables (eTables 1–6) and figures (eFigures 1–4) provide additional methodological detail and validation results supporting the study’s suicide prediction models, including definitions of outcomes, main predictors, handling of missing data, and model evaluation. They also present extended analyses demonstrating model performance, calibration, fairness across populations, and potential clinical utility. Overall, these materials strengthen the transparency, robustness, and applicability of the study’s predictive approach. eTable 1: Suicide‐related Outcomes International Classification of Diseases Clinical Modification (ICD‐10‐CM) Codes. eTable 2: Selected Main Predictors Measured in 6‐month Intervals for Predicting Subsequent Suicide‐related Outcomes. eTable 3: Methods for Missing Value Imputation. eTable 4: Performance Metrics. eTable 5: Summary of Suicide‐related Outcomes by 6‐month Interval in Both Cohorts. eTable 6: Performance Comparison Among Suicide Prediction Models using OneFL 2016–2018 Testing Cohort. eFigure 1: Performance Metrics for Predicting Suicide‐related Outcomes Using Random Forest. eFigure 2: Calibration Plot for the OneFL Internal and External Validation Datasets Using Random Forest (for 20 Population Bins of Equal Size). eFigure 3: Race Discrimination Check for the OneFL Internal and External Validation Datasets Using Random Forest. eFigure 4: Decision Curve Analysis for the OneFL Internal and External Validation Datasets Using Random Forest.

## Data Availability

Data used in this study are available to researchers under the terms of a data use agreement from the OneFlorida Clinical Research Network. The corresponding author has full access to all the data in the study and takes responsibility for the integrity of the data and the accuracy of the data analysis. The data that support the findings of this study are available upon request from the corresponding author. The data are not publicly available due to privacy or ethical restrictions.
