# Supplementary material for: Machine Learning Model for Predicting Suicide Risks Among Patients With Posttraumatic Stress Disorder Who Received Opioids
Source: Depress Anxiety. 2026 Jul 30;2026:5305506. doi: 10.1155/da/5305506 (PMC13420204; doi:10.1155/da/5305506)
Supplement: Supplementary file 1 — Supporting Information The supplementary tables (eTables 1–6) and figures (eFigures 1–4) provide additional methodological detail and validation results supporting the study’s suicide prediction models, including definitions of outcomes, main predictors, handling of missing data, and model evaluation. They also present extended analyses demonstrating model performance, calibration, fairness across populations, and potential clinical utility. Overall, these materials strengthen the transparency, robustness, and applicability of the study’s predictive approach. eTable 1: Suicide‐related Outcomes International Classification of Diseases Clinical Modification (ICD‐10‐CM) Codes. eTable 2: Selected Main Predictors Measured in 6‐month Intervals for Predicting Subsequent Suicide‐related Outcomes. eTable 3: Methods for Missing Value Imputation. eTable 4: Performance Metrics. eTable 5: Summary of Suicide‐related Outcomes by 6‐month Interval in Both Cohorts. eTable 6: Performance Comparison Among Suicide Prediction Models using OneFL 2016–2018 Testing Cohort. eFigure 1: Performance Metrics for Predicting Suicide‐related Outcomes Using Random Forest. eFigure 2: Calibration Plot for the OneFL Internal and External Validation Datasets Using Random Forest (for 20 Population Bins of Equal Size). eFigure 3: Race Discrimination Check for the OneFL Internal and External Validation Datasets Using Random Forest. eFigure 4: Decision Curve Analysis for the OneFL Internal and External Validation Datasets Using Random Forest. [file DA-2026-5305506-s001.docx]

**Supplementary Materials**

**eTable 1.** Suicide-related Outcomes International Classification of Diseases Clinical Modification (ICD-10-CM) Codes

**eTable 2.** Selected Main Predictors Measured in 6-month Intervals for Predicting Subsequent Suicide-related Outcomes

**eTable 3.** Methods for Missing Value Imputation

**eTable 4.** Performance Metrics

**eTable 5.** Summary of Suicide-related Outcomes by 6-month Interval in Both Cohorts

**eTable 6.** Performance Comparison Among Suicide Prediction Models using OneFL 2016–2018 Testing Cohort

**eFigure 1.** Performance Metrics for Predicting Suicide-related Outcomes Using Random Forest

**eFigure 2.** Calibration Plot for the OneFL Internal and External Validation Datasets Using Random Forest (for 20 Population Bins of Equal Size)

**eFigure 3.** Race Discrimination Check for the OneFL Internal and External Validation Datasets Using Random Forest

**eFigure 4.** Decision Curve Analysis for the OneFL Internal and External Validation Datasets Using Random Forest

**eTable 1. Suicide-related Outcomes International Classification of Diseases Clinical Modification (ICD-10-CM) Codes**

| **Suicide-related Outcomes** | **ICD-10-CM code[1]** |
| --- | --- |
| Suicide Attempts | X71-X83, T14.91, T36–T50, T51–T65, T71 where the 6th character of the code = 2) |
| Suicide Ideation | R45.851 |
| Suicide Death | X60-X84, Y10-Y34, Y87.0, and Y87.2 |

1. Hedegaard H, Schoenbaum M, Claassen C, Crosby A, Holland K, Proescholdbell S. Issues in Developing a Surveillance Case Definition for Nonfatal Suicide Attempt and Intentional Self-harm Using International Classification of Diseases, Tenth Revision, Clinical Modification (ICD-10-CM) Coded Data. Natl Health Stat Report. 2018(108):1-19.

**eTable 2. Selected Main Predictors Measured in 6-month Intervals for Predicting Subsequent Suicide-related Outcomes**

| Prescription opioid use | Non-opioid medication use | Sociodemographic | Health status factors | Opioid prescriber-level factors | Regional-level factors |
| --- | --- | --- | --- | --- | --- |
| · Average opioid daily dose in MME · Any fills of opioids SAO, and LAO · Any fills by opioid ingredient and type (e.g., any fentanyl, SAO-type fentanyl, LAO-type fentanyl) · No. unique opioid prescribers … | · Any BZD fills · Any muscle relaxant fills · Any gabapentinoid fills · Any antidepressant fills · Any anti-PTSD medication fills · No. average monthly non-opioid prescriptions · Any naltrexone fills · Received methadone opioid agonist therapy · Any buprenorphine fills · Number of medications used · Multiple medication use (≥3 medications) … | · Age · Gender · Race (White, Black, other) · Ethnicity (Hispanic, Non-Hispanic, Unknown/Missing) · Rurality of resided county (metropolitan vs. non-metropolitan) | · Any outpatient visits · Any ED visits · Any inpatient visits · Prescription opioid overdose · Heroin overdose · Non-opioid drug use disorders · SUD · Alcohol use disorders · Urine drug tests · Counseling · PTSD · Treated PTSD · OUD · Adjustment disorders · Personality disorders · Psychoses · Delusional disorders · Schizophrenia · Mood disorders · Anxiety disorders · Symptoms and signs involving emotional state  · Alcohol-induced mental disorders · Drug-induced mental or sleep disorders · Other mental health disorders · Suicide-related events history · Personal history of self-harm · Osteoarthritis | · Gender of the primary prescriber · Specialty of the primary prescriber · Average monthly opioid prescribing volume · Average monthly patients receiving opioids | · AHRF total health facilities variables · AHRF health professions variables · AHRF resource scarcity variables · AHRF health training program variables · AHRF hospital expenditure, Medicare costs, VA expenditure · AHRF inpatient days/discharges variables · AHRF other health services utilization variables · AHRF census-based variables (e.g., medium household income, employment) · AHRF health insurance status variables · AHRF housing statistics · Area deprivation index county-health ranking variables … |

eTable 2. (Continued)

| Prescription opioid use | Non-opioid medication use | Sociodemographic | Health status factors | Opioid prescriber-level factors | Regional-level factors |
| --- | --- | --- | --- | --- | --- |
|  |  |  | · Back pain  · Neck pain · Headache or migraine · Temporomandibular   disorder pain  · Rheumatoid arthritis  · Abdominal pain or hernia · Chest pain · Kidney or gallbladder stones · Menstrual or genital reproductive pain · Fractures, concussions, or injuries · Fibromyalgia · Internal orthopedic   device implant/graft · Other pain conditions · Surgical procedures   (e.g., ischemic heart diseases) · Musculoskeletal   disorders · Neuropathies (excluding alcoholic, drug, and optic-related) · Ischemic heart disease · HIV/AIDS · Elixhauser index and individual categories … |  |  |
|  |  |  |  |  |  |

**Abbreviations:** AHRF: Area Health Resources Files; BZD: benzodiazepines; HIV/AIDS: human immunodeficiency virus/acquired immunodeficiency syndrome; LAO: long-acting opioids; MME: morphine milligram equivalent; No: Number of; OUD: opioid use disorder; PTSD: post-traumatic stress disorder; SAO: short-acting opioids; SUD: substance use disorders.

**eTable 3. Methods for Missing Value Imputation**

| **Predictor Type** | **Imputation Method^a^** | **Predictors** |
| --- | --- | --- |
| Incurable or chronic conditions | Last observation carried forward | Aggression/impulsivity, anger issues: irritability and anger, problems related to lifestyle, bipolar disorder, symptoms and signs involving emotional state, epilepsy, family history of mental and behavioral disorders, family-related issues, HIV/AIDS, ischemic heart disease, maltreatment syndromes, adjustment disorders, alcohol-induced mental disorders, anxiety disorders, psychiatric disorders, drug-induced mental or sleep disorders, mood disorders, miscellaneous mental health disorders, personality disorders, PTSD, internal orthopedic device implant and graft, fibromyalgia, osteoarthritis, rheumatoid arthritis, mental and behavioral disorders due to psychoactive substance use, post-traumatic stress disorder, schizophrenic disorders, status migrainosus, personal history of self-harm^b^, traumatic brain injury, tinnitus, domestic violence experience or witness, liver diseases, other nonorganic psychosis |
| Curable or acute conditions | Fill zeros | Methadone poisoning, suicide history (any clinical setting)^b^, lack of appetite/appetite issue, alcohol abuse, cannabis use problems, problems related to care-provider dependency, cognitive confusion, delusional disorder, emotional detachment, dizziness, doctor/medical dissatisfaction, fatigue, heroin overdose, lack of exercise, male genital disorders, musculoskeletal disorders, neuropathies, OUD, SUD, non-heroin and non-methadone overdose, abdominal pain or hernia, back pain, pain catastrophizing, chest pain, headache or migraine, fractures, concussion, injuries, neck pain, menstrual/genital reproductive pain, kidney or gall bladder stones, temporomandibular disorder pain, postoperative complications, respiratory diseases, problems related to the social environment, stroke, non-opioid drug use disorders |

**Abbreviations:** HIV/AIDS: human immunodeficiency virus/acquired immunodeficiency syndrome; OUD: opioid use disorder; PTSD: post-traumatic stress disorder; SUD: substance use disorders.

^a^We applied different missing data imputation methods for diagnostic conditions guided by clinical expertise. However, it is challenging to differentiate chronic and acute conditions solely based on ICD-10-CM diagnosis codes. For example, substance use disorders (e.g., cannabis, alcohol, opioids) may be classified as “in early remission” or “in sustained remission”, depending on the last recorded use and treatment history. Similarly, pain conditions such as migraines, back pain, and neck pain can be either acute or chronic, depending on their duration and severity. Indeed, many diagnoses don’t necessarily carry forward in medical records, particularly if the attending physician does not address the specific condition during a visit. As a result, the absence of a recorded diagnosis does not necessarily indicate that the patient is no longer experiencing symptoms or that they no longer meet the criteria for a given condition. This limitation is especially relevant for conditions categorized as “curable or active”, where a lack of documentation does not imply resolution but rather an absence of explicit reporting in that particular encounter.

^b^Personal history of self-harm was defined by ICD-10-CM code Z91.5. Suicide history (any clinical setting) included suicide attempts, suicide ideation, and suicide death.

**eTable 4. Performance Metrics**

| Metrics | Formula | Description |
| --- | --- | --- |
| PPV (Precision) | $\frac{TP}{TP+FP}$ | Measures the probability that patient cases truly have suicide-related outcomes. PPV is influenced by the prevalence of the outcome of interest. |
| NPV | $\frac{TN}{TN+FN}$ | Measures the probability that health cases truly do not have suicide-related outcomes. When the outcome is rare, NPV is typically high. |
| Sensitivity (Recall) | $\frac{TP}{TP+FN}$ | Measures the ability to determine patient cases correctly. |
| Specificity | $\frac{TN}{TN+FP}$ | Measures the ability to determine health cases correctly. |
| F-1 Score | $\frac{2TP}{2TP+FP+FN}$ | The harmonic mean of the precision and recall. |
| Accuracy | $\frac{TP+TN}{TP+TN+FN+FP}$ | Measures the ability to make correct predictions. |
| Weighted Accuracy | $\frac{5TP+TN}{5TP+TN+5FN+FP}$ | Assuming that a TP event is rewarded 5 times more than a TN event, and a FN event is penalized 5 times more than a FP event. |
| C-statistic | Sensitivity vs. (1-Specificity) | The area under the ROC curve is a plot of sensitivity vs. (1-specificity) for all potential cut-off probability thresholds for an algorithm. Comparisons of C-statistics based on imbalanced data or rare outcomes can be misleading because C-statistics do not incorporate information about the prevalence or pre-test probability of the outcome. |
| Precision-recall curves | PPV vs. Sensitivity | A precision-recall curve of precision (PPV; y-axis) vs. recall (sensitivity; x-axis). The curve closer to the upper right corner (corresponding to 100% precision and 100% recall) has better performance. |

**Abbreviations:** FN: false negative (the number of cases incorrectly identified as healthy), FP: false positive (the number of cases incorrectly identified as a patient), TN: true negative (the number of cases correctly identified as healthy), TP: true positive (the number of cases correctly identified as a patient), PPV: positive predictive value, NPV: negative predictive value, ROC: receiver operating characteristics.

**eTable 5. Summary of Suicide-related Outcomes by 6-month Interval in Both Cohorts**

| **OneFL EHR and Florida Medicaid linked data** | **Number of Patients** | **Patients with IP/ED Suicide-Related Outcomes^a^** | **6-Month Suicide Rate (%)** |
| --- | --- | --- | --- |
| **2016–2018 Training (N=3718)** |  |  |  |
| Interval 0 (Baseline) | 3718 | 288 | 7.7 |
| Interval 1 | 3089 | 182 | 5.9 |
| Interval 2 | 2143 | 115 | 5.4 |
| Interval 3 | 1358 | 80 | 5.9 |
| Interval 4 | 610 | 29 | 4.8 |
| Total | 10918 | 694 | 6.4 |
| **2016–2018 Testing (N=1860)** |  |  |  |
| Interval 0 (Baseline) | 1860 | 154 | 8.3 |
| Interval 1 | 1546 | 85 | 5.5 |
| Interval 2 | 1022 | 53 | 5.2 |
| Interval 3 | 626 | 39 | 6.2 |
| Interval 4 | 302 | 15 | 5.0 |
| Total | 5356 | 346 | 6.5 |
| **2019–2021 Validation (N=4849)** |  |  |  |
| Interval 0 (Baseline) | 4849 | 302 | 6.2 |
| Interval 1 | 4245 | 209 | 4.9 |
| Interval 2 | 3010 | 130 | 4.3 |
| Interval 3 | 1918 | 68 | 3.5 |
| Interval 4 | 1017 | 34 | 3.3 |
| Total | 15039 | 743 | 4.9 |

**Abbreviations:** ED: emergency department, EHR: electronic health records, IP: inpatient.

^a^IP/ED Suicide-related outcomes observed in the subsequent 6-month interval.

**eTable 6. Performance Comparison Among Suicide Prediction Models using OneFL 2016–2018 Testing Cohort**

| Performance Metrics | RF^a^ | LASSO | GBM | DNN |
| --- | --- | --- | --- | --- |
| TP-FP-FN-TN  (total number of intervals) | 225-620-121-4390 (5356) | 229-957-117-4053 (5356) | 209-753-137-4257 (5356) | 244-792-102-4218 (5356) |
| F1 score | 0.38 | 0.30 | 0.32 | 0.35 |
| C-statistic | 83.6% | 81.7% | 83.4% | 84.2% |
| Specificity | 87.6% | 80.9% | 85.0% | 84.2% |
| Sensitivity (Recall) | 65.0% | 66.2% | 60.4% | 70.5% |
| Positive predictive value (Precision) | 26.6% | 19.3% | 21.7% | 23.6% |
| Negative predictive value | 97.3% | 97.2% | 96.9% | 97.6% |
| Accuracy | 86.2% | 80.0% | 83.4% | 83.3% |
| Weighted Accuracy^b^ | 81.8% | 77.1% | 78.7% | 80.7% |

**Abbreviations:** FN: false negative (the number of cases incorrectly identified as healthy), FP: false positive (the number of cases incorrectly identified as a patient), TN: true negative (the number of cases correctly identified as healthy), TP: true positive (the number of cases correctly identified as a patient), PPV: positive predictive value, NPV: negative predictive value, LASSO: least absolute shrinkage and selection operator regression, GBM: gradient boosting machine, RF: random forest, DNN: deep neural networks.

^a^We selected RF as the final model because it has the best F-1 score, best weighted accuracy, high C-statistics, relatively high sensitivity, and fast computing time in the testing data among all the machine learning approaches. Yet RF, LASSO, GBM, and DNN all have low precision and overfitting issues due to the low prevalence rate of suicide-related outcomes. DNN gives a similar performance as RF, but it is much more time-consuming than other models.

^b^Assuming that a TP event is rewarded 5 times more than a TN event, and an FN event is penalized 5 times more than an FP event.

**eFigure 1. Performance Metrics for Predicting Suicide-related Outcomes Using Random Forest**

| A ROC (primary: 6mo/6mo IP/ED suicide)  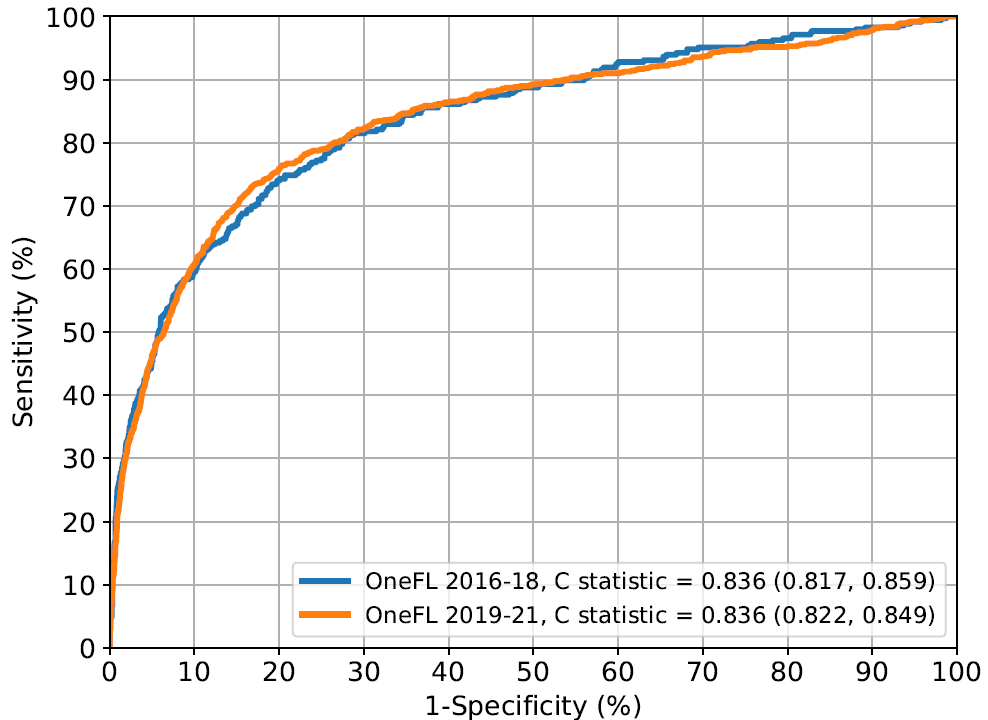 | B Precision-recall (primary: 6mo/6mo IP/ED suicide)  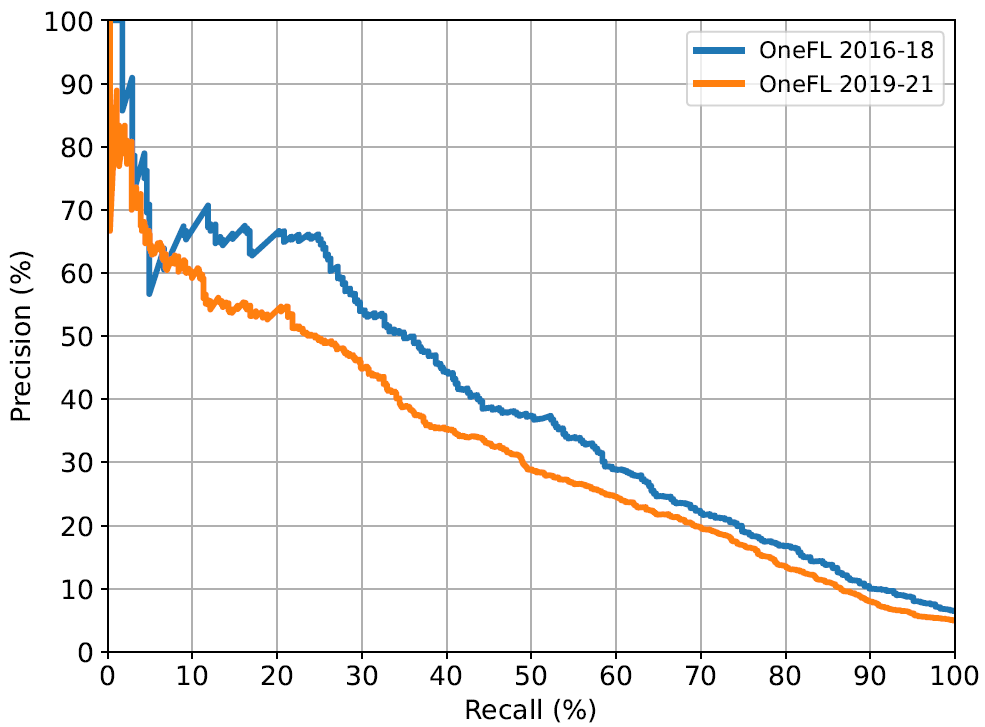 |
| --- | --- |
| C ROC (6mo/3mo IP/ED suicide)  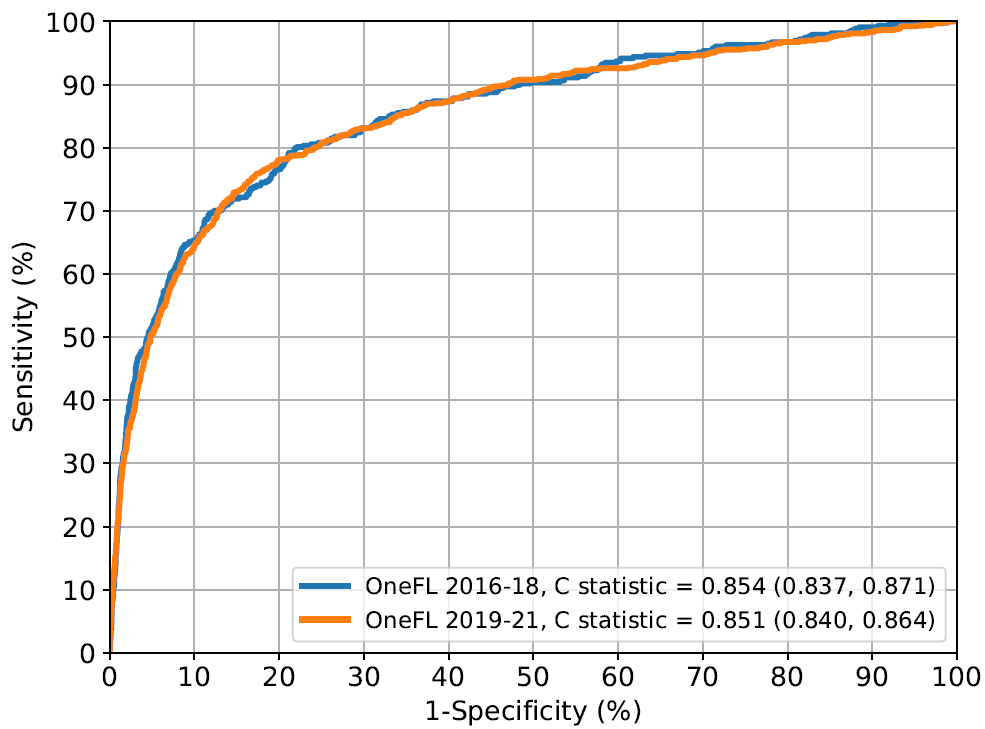  E ROC (6mo/6mo IP/ED/OP suicide)  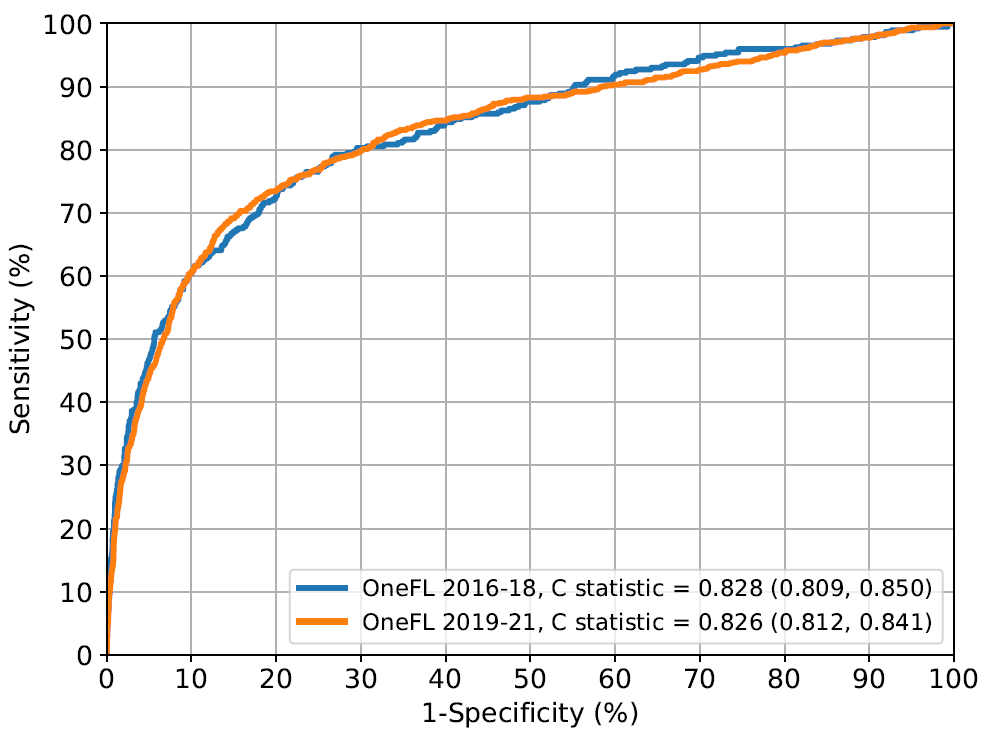 | D Precision-recall (6mo/3mo IP/ED suicide)  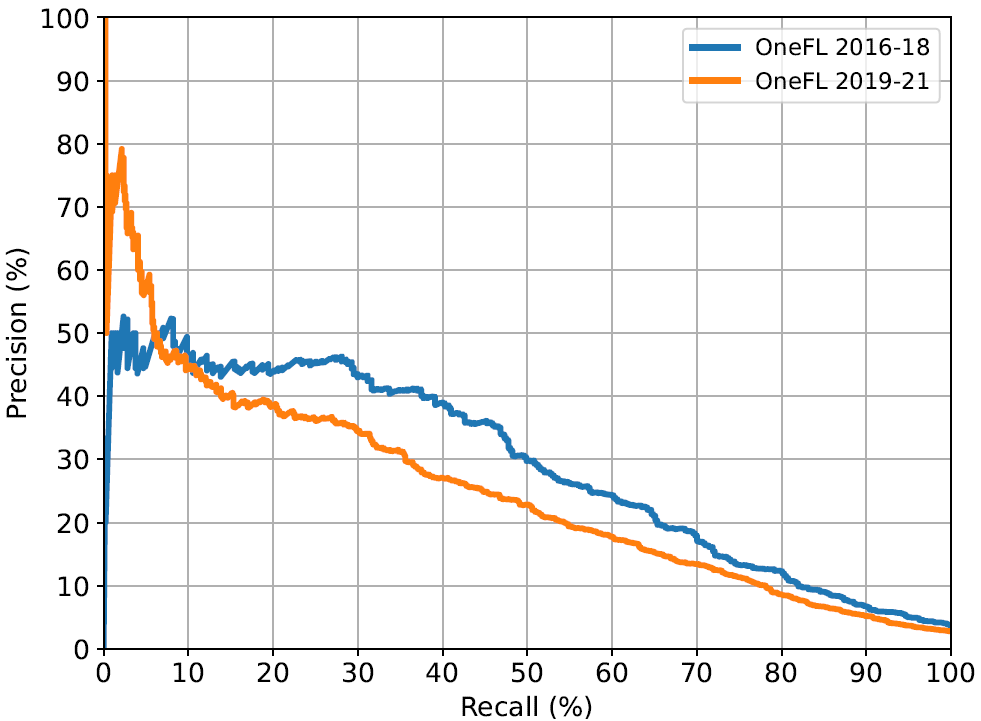  F Precision-recall (6mo/6mo IP/ED/OP suicide)  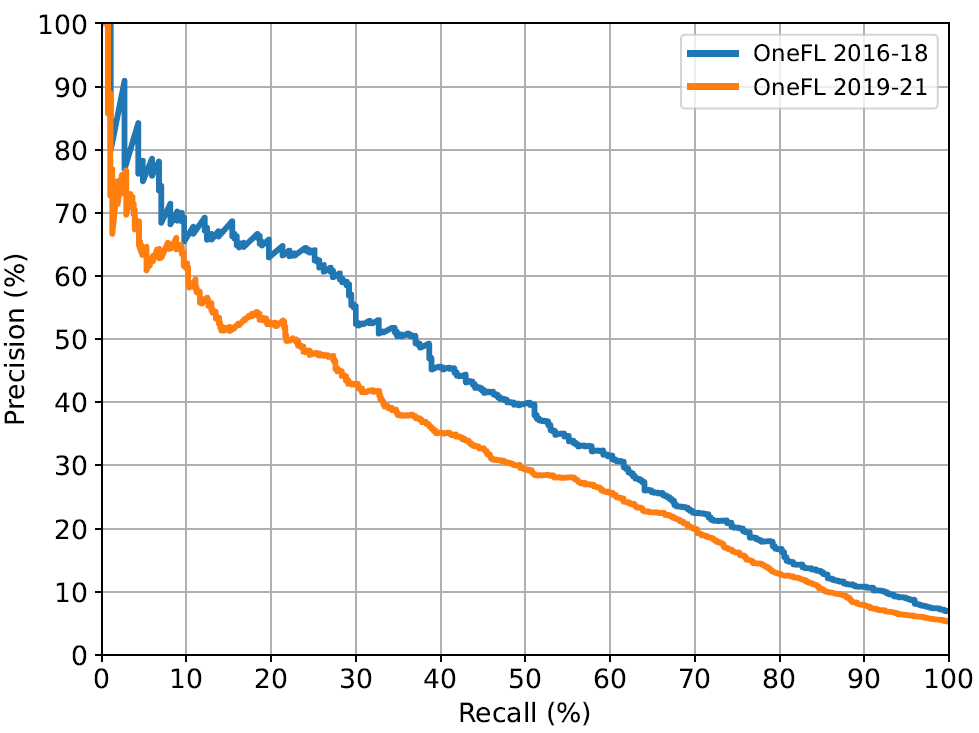 |

**Abbreviations:** ED: emergency department, IP: inpatient, mo: month, OP: outpatient, ROC: receiver operating characteristic curve.

**eFigure 2. Calibration Plot for the OneFL Internal and External Validation Datasets Using Random Forest (for 20 Population Bins of Equal Size)**

**
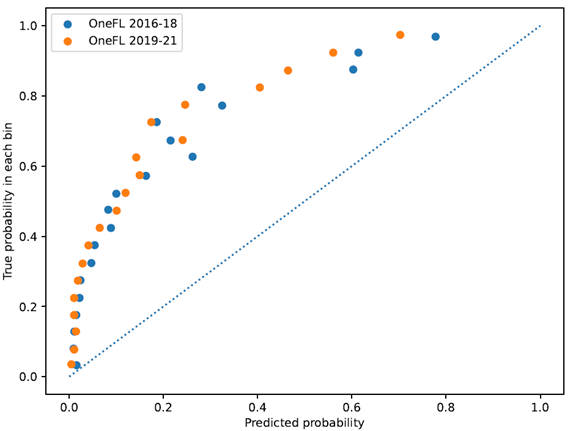
**

Our random forest model tends to overestimate suicide risks because it predicts a probability of suicide-related outcomes higher than the actual event probability. If the model is perfectly calibrated, the points would lie along the diagonal line. For example, with a predicted probability of 0.7 for a suicide-related outcome event, the actual observed frequency of the event should occur about 70% of the time in the bin.

**eFigure 3. Race Discrimination Check for the OneFL Internal and External Validation Datasets Using Random Forest**

| A FNR by race (OneFL 2016–18 internal)  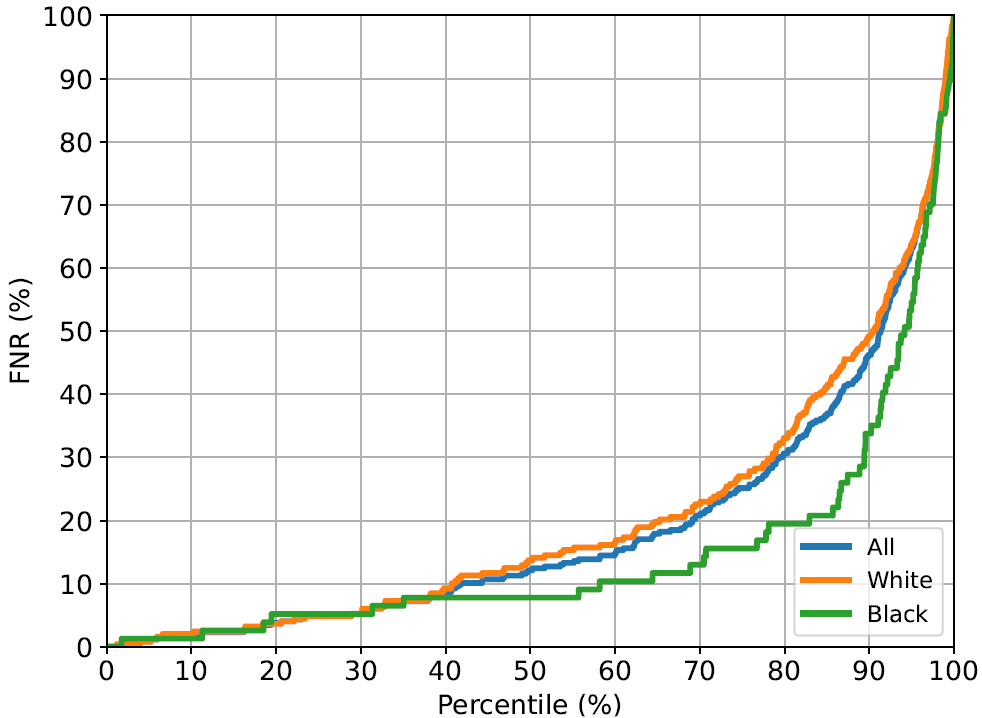 | B FPR by race (OneFL 2016–18 internal)  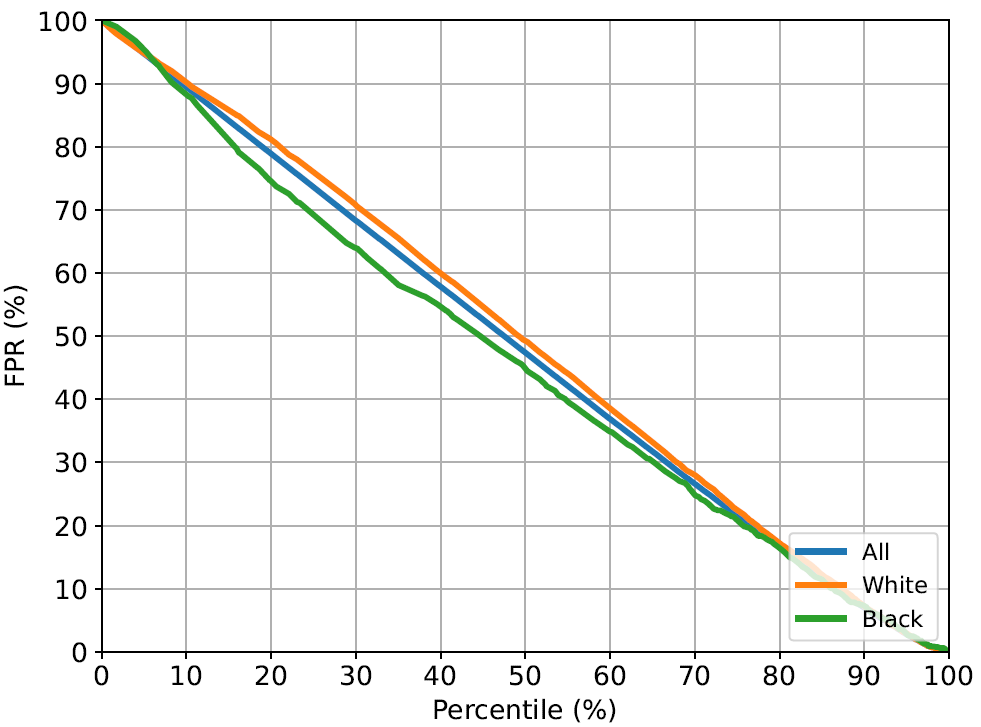 |
| --- | --- |
| C ROC by race (OneFL 2016–18 internal)  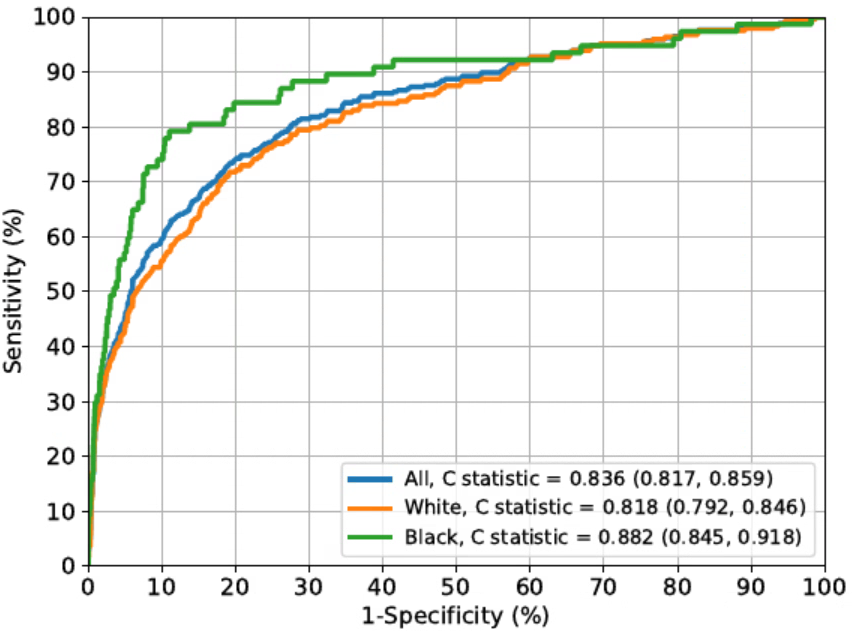 | D Precision-recall by race (OneFL 2016–18 internal)  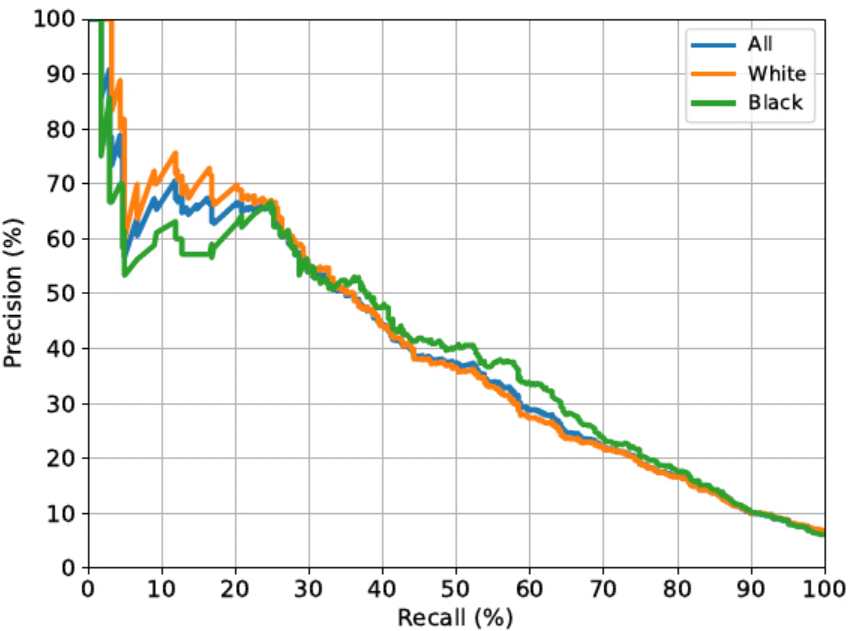 |
| E FNR by race (OneFL 2019–21 external) 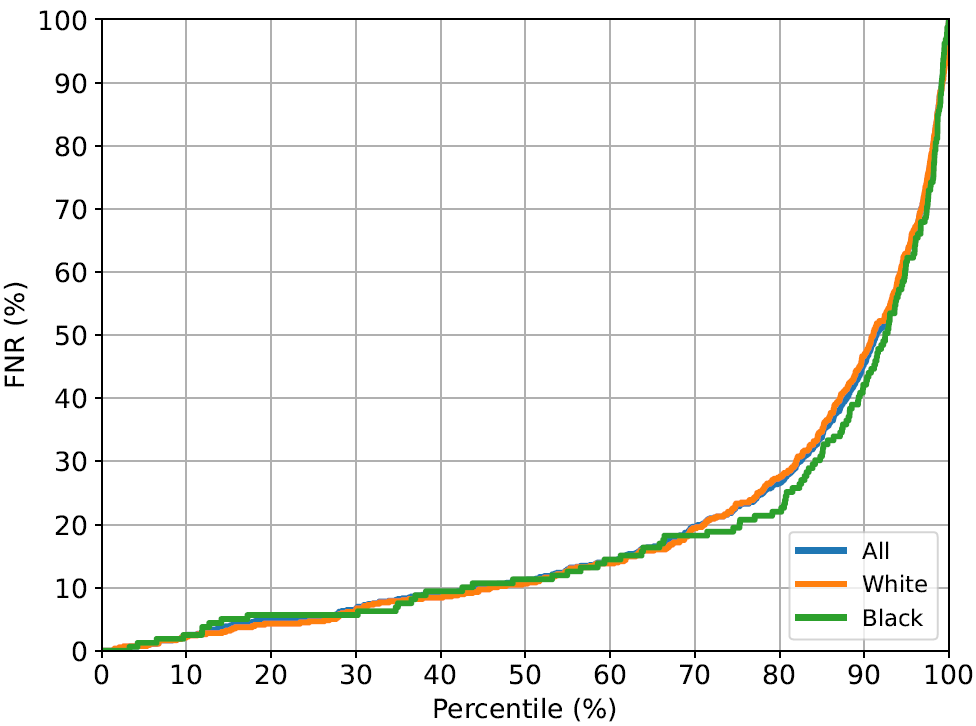 | F FPR by race (OneFL 2019–21 external)  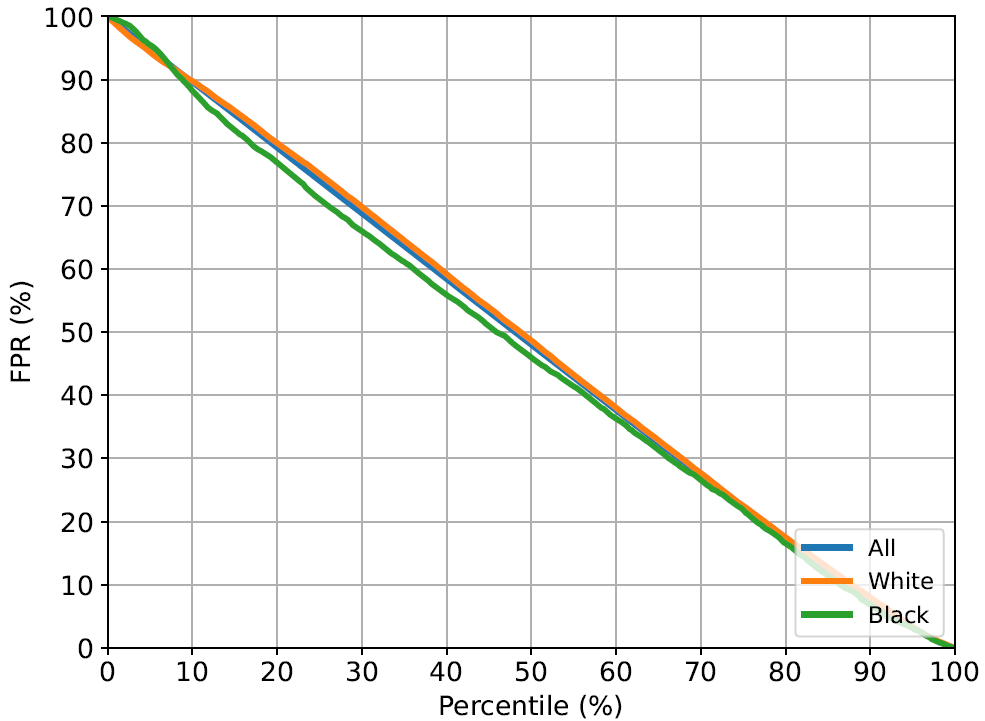 |
| G ROC by race (OneFL 2019–21 external)  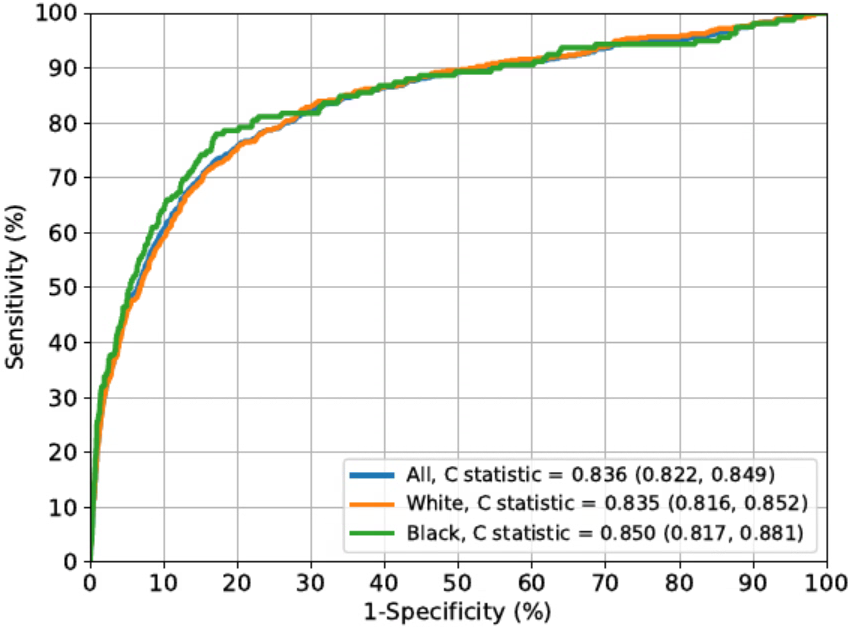 | H Precision-recall by race (OneFL 2019–21 external)  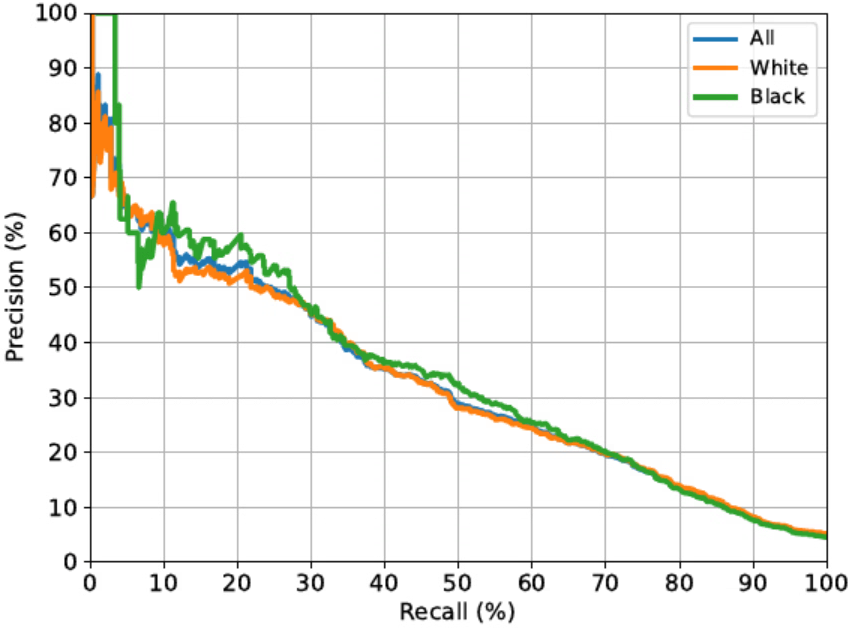 |
| I Calibration plot by race (OneFL 2016–18 internal)  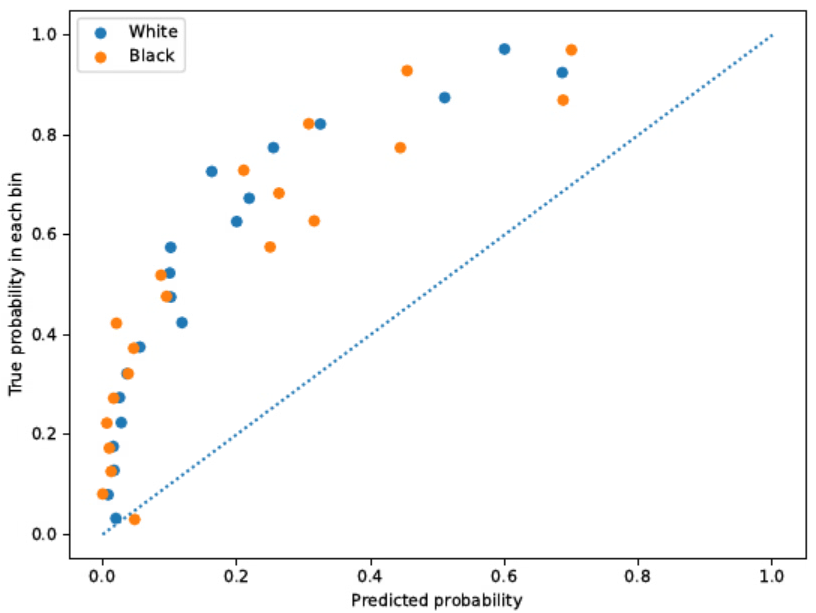 | J Calibration plot by race (OneFL 2019–21 external)  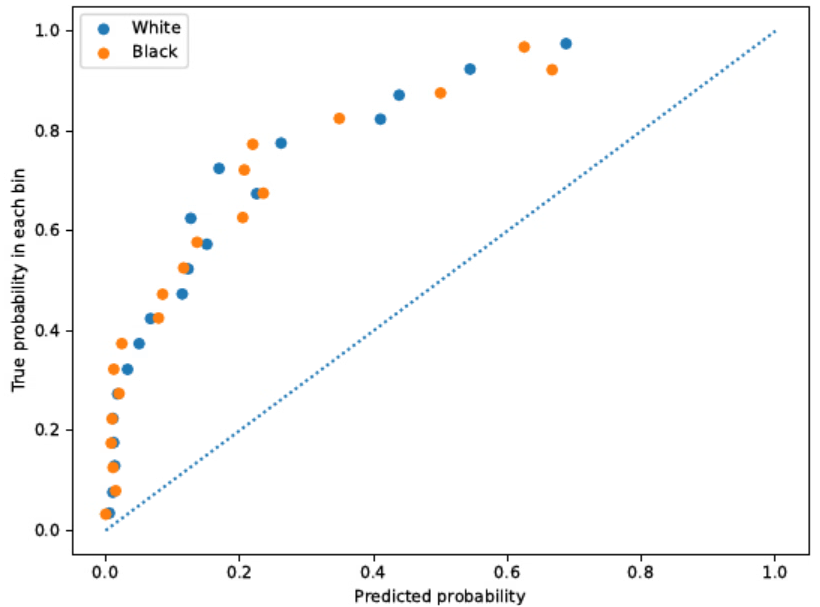 |

**Abbreviation:** FNR: false negative rate, FPR: false positive rate, ROC: receiver operating characteristic curve.

**eFigure 4. Decision Curve Analysis for the OneFL Internal and External Validation Datasets Using Random Forest**

| A DCA curve (OneFL 2016–18 internal)  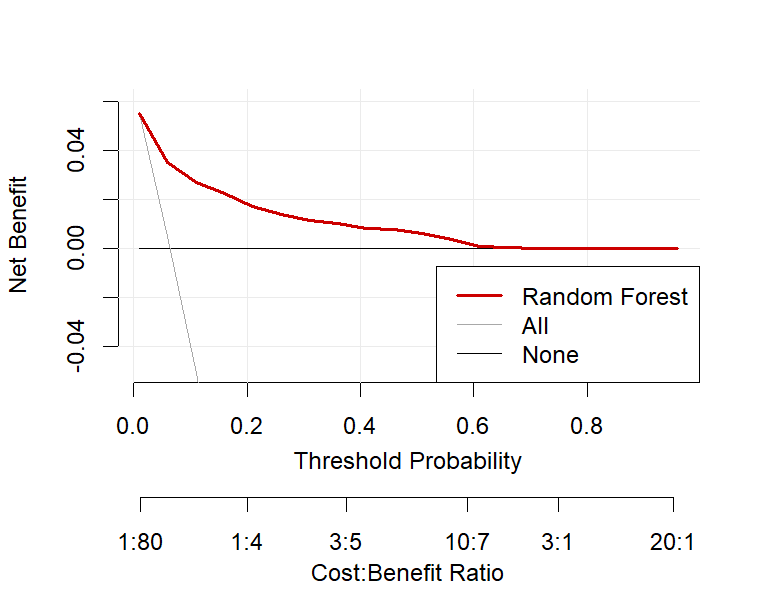 | B DCA curve (OneFL 2019–21 external)  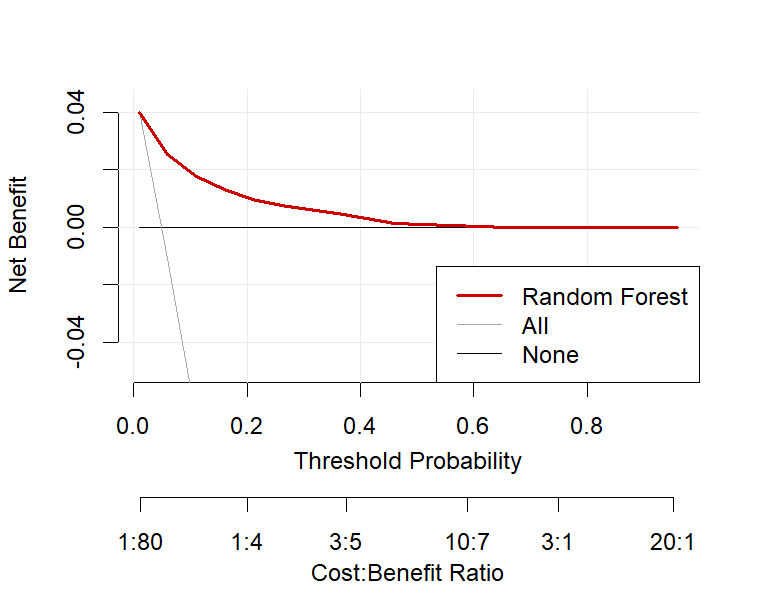 |
| --- | --- |

**Abbreviation:** DCA: decision curve analysis.

This decision curve analysis demonstrated that our random forest model provides meaningful clinical utility across threshold probabilities ranging from approximately 1% to 60%. Across this range, the model’s net benefit remains consistently positive and exceeds that of both treat-all and treat-none strategies. At lower thresholds (e.g., 1–5%), which are particularly relevant in suicide prevention where missing a high-risk individual carries substantial consequences, the model yields the greatest net benefit. This indicates that using the model to guide intervention decisions would identify more individuals at true risk of suicide while avoiding unnecessary interventions compared with a strategy of intervening on all individuals.

As the threshold probability increases, reflecting more conservative decision-making or greater concern about over-intervention, the model’s net benefit gradually declines but remains superior to both alternative strategies. In contrast, the treat-all strategy becomes increasingly harmful (negative net benefit) as thresholds rise, reflecting the clinical and resource burden associated with unnecessary monitoring or intervention among low-risk individuals. The treat-none strategy, by definition, provides zero net benefit and would fail to identify individuals at elevated risk.
